# Revolutionizing electrocardiography: the role of artificial intelligence in modern cardiac diagnostics

**DOI:** 10.1097/MS9.0000000000002778

**Published:** 2025-01-09

**Authors:** Sardar N. Qayyum, Muhammad Iftikhar, Muhammad Rehan, Gulmeena Aziz Khan, Maleeka Khan, Risha Naeem, Rafay S. Ansari, Irfan Ullah, Samim Noori

**Affiliations:** a Department of Internal Medicine, Bacha Khan Medical College, Mardan, Pakistan; b Khyber Medical College, Peshawar, Pakistan; c Department of Internal Medicine, Al-Nafees Medical College and Hospital, Islamabad, Pakistan; d Ameer-ud-din Medical College, University of Health Sciences, Lahore, Lahore, Pakistan; e Ziauddin Medical University, Pakistan; f Nangarhar University Faculty of Medicine, Nangarhar, Afghanistan

**Keywords:** electrocardiogram, artificial intelligence, machine learning, deep learning, ECG databases, precision cardiology

## Abstract

Electrocardiography (ECG) remains a cornerstone of non-invasive cardiac diagnostics, yet manual interpretation poses challenges due to its complexity and time consumption. The integration of Artificial Intelligence (AI), particularly through Deep Learning (DL) models, has revolutionized ECG analysis by enabling automated, high-precision diagnostics. This review highlights the recent advancements in AI-driven ECG applications, focusing on arrhythmia detection, abnormal beat classification, and the prediction of structural heart diseases. AI algorithms, especially convolutional neural networks (CNNs), have demonstrated superior accuracy compared to human experts in several studies, achieving precise classification of ECG patterns across multiple diagnostic categories. Despite the promise, real-world implementation faces challenges, including model interpretability, data privacy concerns, and the need for diversified training datasets. Addressing these challenges through ongoing research will be crucial to fully realize AI’s potential in enhancing clinical workflows and personalizing cardiac care. AI-driven ECG systems are poised to significantly advance the accuracy, efficiency, and scalability of cardiac diagnostics.

## Introduction

Artificial Intelligence (AI) makes machines capable of simulating intelligence and endows them with human-like capabilities such as understanding, reasoning, decision-making, problem-solving and performing complex tasks with varying degrees of autonomy^[1]^. In healthcare, AI-powered technologies have been used for analysis and interpretation of various medical images such as X-rays, CT-scan for diagnosis and risk predictions. Various AI algorithms have been developed and improved over time for ECG analysis as well^[2]^.

**Figure 1.**
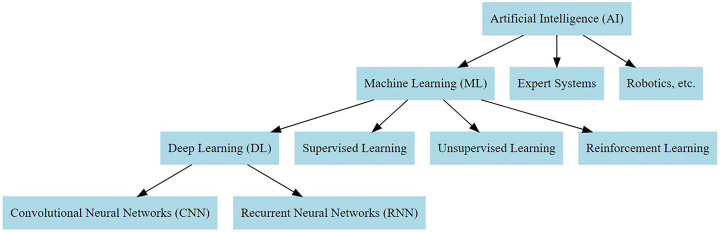
The relationship between AI, ML, and DL.

Machine learning (ML), a subtype of AI, focuses on the development of algorithms and statistical techniques that enable computer systems to learn from the datasets, identify patterns and predict outcomes based on the input data. ML approaches include supervised learning, unsupervised learning, reinforcement learning and deep learning (DL)^[3]^. In supervised learning, algorithms are trained on labeled datasets to predict outcomes on new, unseen data while in unsupervised learning, algorithm learns patterns in datasets to predict outcomes in new data without human supervision. Reinforcement learning makes computer systems to learn to make decisions and solve problems by themselves based on a reward and penalty systems as in robotics. Deep learning (DL) is a subset of ML, which uses neural networks to learn from large datasets and extract hidden patterns. These multilayered neural networks consist of interconnected nodes organized in layers that mimic human brain in structure and function. DL requires large datasets to train effectively and can also learn automatically from unstructured data unlike ML which often requires manual feature extraction and selection. DL networks, such as CNNs (convolutional neural networks), has feature extraction layers also known as convolutional filters which automatically extracts features from the data and model layers to analyze the extracted features^[4]^. Thus, DL models learn features relevant to the task directly from the data avoiding human bias and the need for manual feature engineering^[1]^. Figure 1 summarizes the relationship of AI, ML, and DL.

AI has been adopted to analyze routine 12-lead ECGs after being widely used in computer-vision, image processing and speech recognition. AI applications in ECG analysis include simple rule-based models to state-of-the art DL models that can mimic human-like interpretation. The DL models such as CNN can learn from temporal information to detect voltage variations or spatial information to locate myocardial infarction^[5]^. CNNs are the most widely used models in AI-ECG systems for tasks like arrhythmia detection, abnormal beat classification, and structural heart disease prediction. CNNs excel at learning spatial and temporal patterns in ECG signals, which makes them ideal for analyzing ECG data with time-series characteristics. CNNs involve operations such as “convolutions” and “pooling,” which can become computationally expensive, especially when applied to long ECG recordings with multiple leads. The computational complexity increases significantly as the number of filters or inputs size grows, especially when applied to 12-lead ECGs^[6]^.

Artificial intelligence (AI) has emerged as a transformative force across various medical specialties, revolutionizing both clinical support and data interpretation. In radiology, AI is being harnessed to enhance diagnostic precision, with continuous learning algorithms enabling faster and more accurate detection of abnormalities in imaging studies such as CT scans and MRIs^[5]^. In oncology, AI-driven models are being utilized from early cancer detection to personalized treatment planning, improving patient outcomes through tailored therapies. Similarly, in cardiology, AI is being integrated into echocardiogram analysis, enabling automated detection of heart abnormalities and predictive analytics for future cardiovascular events. Dentistry is also benefiting from AI, particularly in dental implant planning, where machine learning algorithms optimize treatment strategies based on patient-specific data. Moreover, in specialties such as pathology, AI is assisting in the analysis of tissue samples, improving diagnostic accuracy and reducing human error.^[2]^

**Figure 2.**
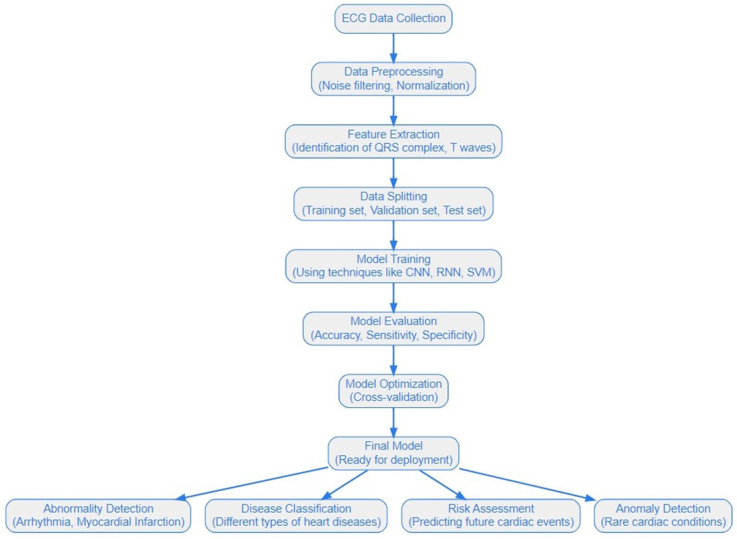
The functioning of AI-ECG systems.

In this review, we aim to address the following research question: How can AI, particularly deep learning models, be utilized to enhance the accuracy, efficiency, and diagnostic capabilities of electrocardiogram (ECG) analysis, with a specific focus on arrhythmia detection, structural cardiac abnormalities, and long-term cardiac monitoring? Figure 2 shows the working of AI algorithms in ECG analysis termed “AI-ECG systems.”

## Methodology

We searched the last 5 years literature on databases such as PubMed, Scopus, Google scholar using search terms such as “Artificial Intelligence,” “Deep learning,” “Convolutional Neural Networks (CNN),” and “Electrocardiogram,” “ECG,” “Arrhythmia Detection,” “Abnormal Beats,” “Atrial Fibrillation,” and “Structural Heart diseases.” We initially screened 70 studies published in peer-reviewed journals. We included papers published within last 5 years that focused on the applications of AI, ML, and DL in the analysis and interpretation of ECG. Studies not related to AI or ECG, or published before 2018, non-peer-reviewed articles, opinions, pieces, or commentaries were excluded. Studies not available in English were also excluded. We included a total of 51 studies focusing on the applications of AI, ML, and DL on ECG analysis and interpretation with special focus on arrhythmia detection, structural cardiac abnormalities, and long-term cardiac monitoring.

## A review of applications of AI in ECG analysis

### Technical specifications of major ECG datasets

The ECG datasets have a spatial axis which refers to voltage recordings of different leads measuring the heart’s electrical activity from different angles and a temporal axis which reflects changes in voltage over time. The technical specifications of major ECG datasets have been given in Table 1.

**Table 1:** Technical specifications of various important ECG databases

Dataset	Content description	Channels	Duration	Annotations
MIH-BIH Data	48 half-hour excerpts of 2-channel ambulatory ECG recordings from 47 subjects	2 leads	Each recording is 30 minutes long	Beat annotations including types and location of arrhythmias
PhysioNet Computing in Cardiology (CinC) Challenge dataset	Annual challenge datasets with varying content	Varies from 1 to 12 leads	10 s to several minutes	Specific to each year’s challenge; often include arrhythmia types, signal quality indicators, etc.
PTB Diagnostic ECG Database	549 records from 290 subjects, including healthy controls and patients with various conditions	15 leads including 12 leads + 3 Frank leads	Each recording is approx. 2 min long	Clinical diagnoses and signal quality indicators
St. Petersburg INCART Database	75 annotated recordings 32 Holter monitor files of 32 subjects	12 leads	Each recording is 30 minutes long	Beat annotations, rhythm changes, and arrhythmias
Chapman Shaoxing and Ningbo Hospital database	Over 45 000 ECG recordings	12 leads	Each recording is 10 s long	Detailed annotations include rhythm types, noise levels, and clinical findings
Lobachevsky University Electrocardiography Database	Contains 200 ECG recordings	12 leads	Each recording is 10 s long	Beat annotation, rhythm changes, and clinical annotations
Georgia 12-lead ECG challenge Database	Over 10 000 12-lead ECGs used in PhysioNet 2020	12 leads	Each recording is 10 s to 2 min long	Beat annotation, rhythm classification, and clinical diagnoses
Long-term ST database	86 long-term ECG recordings of 80 subjects	2 or more leads	Each recording is 21 to 24 h long	ST segment annotations and ischemic events
European ST-T database	90 annotated ECG recordings from 79 subjects	2 leads	Each recording is 2 h long	Annotations of ST-T segment changes
CSE Database	Standard ECG database for validation of AI algorithms	12 leads	Each recording is 10 s long	Clinical annotations and diagnostic markers
QT Database	105 ECG recordings annotated with QT intervals	2 leads	Each recording is 15 min long	QT interval annotations and arrhythmia markers
Mayo clinic database	Over 10 million ECG recordings	12 leads	Each recording is 10 s long	Beat annotation, rhythm classification, clinical diagnoses, etc.

### AI for abnormal beat detection

Abnormal Heartbeats can either be ectopic beats and arrhythmias when they form a specific pattern. Although ectopic beats have less clinical relevance for healthy patients but in CVD patients may have more significance and can be indicative of higher arrhythmia risks^[7]^. Classification AI models make use of supervised data mining technique wherein a model is trained on labeled ECG datasets in which input data is paired with correct output. During the training phase, the model learns to associate input features with specific categories and distinguish between normal and abnormal beats and rhythms. This supervised approach ensures that the AI systems can accurately categorize new, unseen ECG data, thereby enhancing diagnostic precision and potentially identifying cardiac abnormalities with greater reliability^[8]^. DL algorithms are well-suited for complex datasets and their performance also improves with exposure to larger datasets. DL models achieved higher performance metrics than other AI techniques in several studies. A DL approach, 1D-CNN, has been developed in a study by Malik *et al*^[8]^ to detect ectopic beats such as ventricular and supra-ventricular beats. The model achieved an accuracy of 98% and 99.04% for supra-ventricular and ventricular ectopic beats respectively. In another study, Li *et al*^[9]^ developed GRNN (general regression neural network) for beat classification and the model achieved an accuracy of up to 95%. A CNN model^[10]^ was developed which could classify heartbeats into five classes, i.e., supraventricular ectopic, ventricular ectopic, non-ectopic, fusion and unknown beats. The model achieved an accuracy of above 90%. Noises in datasets may lead to misinterpretation and misdiagnosis thus the accuracy of AI model declined to 89% in noisy ECGs. Noises in ECG data can arise from various sources, such as muscle activity, baseline wander, motion artifacts, power-line interference, and electrode displacement. CNN models will perform well with minor noise, but their performance degrades as noise increases because CNNs rely heavily on spatial feature extraction, and noise in the spatial domain can corrupt features used for classification such as QRS complex, P wave, and T wave. 1D-CNNs can also be affected by noise, but they may show better resilience due to their focus on temporal data. However, when the noise is persistent across time-series data, the model may struggle to distinguish between signal and noise. GRNNs tends to be highly sensitive to noisy data because they are non-parametric models that use entire training set for predictions, any noise in the dataset can propagate into model’s predictions.^[11]^

Another study developed a semi-supervised model by using DL approach Gaussian-Bernoulli deep belief network (GB-DBN) along with an SVM classifier^[12]^. The model was trained, tested and validated across two MIT-BIH databases for the classification of SVEB and VEB. DBN and SVM are hybrid models useful when labeled data is scarce, but these models tend to be computationally heavy during training especially when applied to large-scale ECG datasets, but are less computationally intensive than CNN-based models^[13]^. The proposed model^[12]^ works in three phases. In the first phase, the GB-DBN learns the feature representation in the datasets followed by SVM classifier’s learning the classification. The last phase is characterized by an active learning phase where it interacts with an expert for ambiguous beats classification. Initially, the model achieved 91.3% sensitivity, 98.52% specificity, and 98.1% overall accuracy for VEB which improved to 98.47% sensitivity, 99.5% specificity, and 99.42% accuracy after multiple queries with experts. Similarly, the model achieved an initial sensitivity of 38.8%, 99.2% specificity, and 97.5% accuracy for SVEB which improved to 94.3% sensitivity, 99.71% specificity, and 99.6% overall accuracy. In another study, a rule-based system was developed to detect ischemic and arrhythmic heartbeats^[14]^. The rule-based model achieved 91% sensitivity and 92% specificity for an ischemic beat while 96% sensitivity and 99% specificity for all other categories indicating the proposed model could be applied to clinical settings for ECG analysis. In the above studies, AI has demonstrated the feasibility of fast, accurate and personalized beat classification. These models are useful for patient’s ECG data requiring long-term cardiac monitoring for abnormal beat detection with higher accuracy.

### AI for arrhythmia detection

AI algorithms focused on the analysis of the broader pattern of irregular heart activity over time have been developed for the detection and classification of arrhythmias such as atrial fibrillation (AFib), bradycardia, tachycardia, and ventricular fibrillation, with higher or comparable accuracy than cardiologists^[15]^.

### AFib detection

AFib is the most common type of arrhythmias and is mostly asymptomatic until late complications such as thrombosis, stroke, and heart failure. Attia *et al*^[16]^ developed a CNN model to analyze standard 12 ECGs during normal rhythm and identify patients with history of AFib. The model was trained with over 650 000 ECGs from the Mayo Clinic Database and tested on dataset of over 35 000 patients. Initially the model achieved an AUC of 0.87, 79% sensitivity, 80% specificity and 79% accuracy. However, as the performance of DL models improve with time, so after 1 month the performance of the DL model increased. It achieved an AUC of 0.90 with sensitivity of 82%, specificity and accuracy of 83%. AI offers a rapid, inexpensive way to detect patients with undetected AFib using standard ECGs during normal sinus rhythm unlike to conventional techniques that involve long-term cardiac monitoring. In another study, Raghunath *et al*^[17]^ developed a deep neural network to predict new-onset of AFib within 1 year. The algorithm was trained on a larger dataset of over 1.6 million standard ECGs obtained from 253,387 patients. The training dataset included 99,371 patients with previous AFib episodes. The model was evaluated on a test set of 168,914 patients, in which 14,207 AFib episodes had occurred and it achieved an AUC of 0.88. Thus, the application of DL includes not only detection of AFib but also identification of patients at greater risk of recurrence.

### Ventricular arrhythmia (VA) detection

Ventricular arrhythmias such as ventricular tachycardia (VT) and premature ventricular complexes (PVC) result into the deterioration of LV ejection fraction. Rapid detection and application of defibrillators are crucial for treating life-threatening arrhythmias^[18]^. Sabut *et al*^[19]^ trained, tested and validated a DNN model on two public-domain ECG databases. The model achieved better performance than any standard ML-based classifier, i.e., an accuracy of 99.2% with sensitivity of 98.8% and specificity of 99.3%. Another study applied the CNN model to locate the source of VA from standard 12 ECGs achieving an AUC of 0.963 for binary classification as right or left VA and an AUC of 0.998 for detecting VA originating from LV summit. The proposed DL algorithms could not only detect VTA accurately but also locate the VTA source improving the efficiency of cardiac diagnosis and guiding catheter ablation.

### AI as a unified solution for multiclass arrhythmia classification

An ensemble model, STA-CRNN, developed by Zhang *et al*^[20]^ classified standard ECGs into normal heart rhythms and 8 classes of arrhythmias including AFib, 1-AVB, LBBB, RBBB, PAC, PVC, ST-segment depression and ST-segment elevation. The model’s architecture was based on CNN’s local feature extraction and RNN’s global feature extraction abilities and the spatiotemporal attention module compensates for the noise in the dataset. The classification model was trained on the CPSC database and achieved an average F1 score of 0.835. Several of the AI classification models can classify multi-lead ECGs into 20 or more diagnostic classes. Zhu *et al*^[21]^ developed an ensemble model by combining a rule-based system and two SE_ResNet (an ANN) models to classify 27 cardiac abnormalities including AFib, AFL, bradycardia, bundle branch blocks, PVC, prolonged QT interval, prolonged PR interval, sinus arrhythmias, T-wave abnormalities, etc. The initial framework of SE_ResNet is based on CNN architecture. The sign loss function is used in addition to dealing with noise in data and improving the generalizability. It was trained and tested on 6 different PhysioNet datasets and ranked 3rd in the PhysioNet/Computing in Cardiology Challenge 2020. Xu *et al*^[22]^ developed a more robust DL model to classify ECGs into 30 cardiac abnormalities The model was trained, validated and trained on PhysioNet dataset and achieved higher accuracy AUC (> 0.90) for sinus bradycardia, sinus tachycardia, AFL, LBBB, RBBB, IAVB, LAD, LAnFB, NSR, etc., using standard 12-Lead ECGs and 2-Lead ECGs. These models are recommended for use in clinical settings to detect multiple classes of arrhythmias.

### AI as high precision tool for multiclass arrhythmia classification

**Figure 3.**
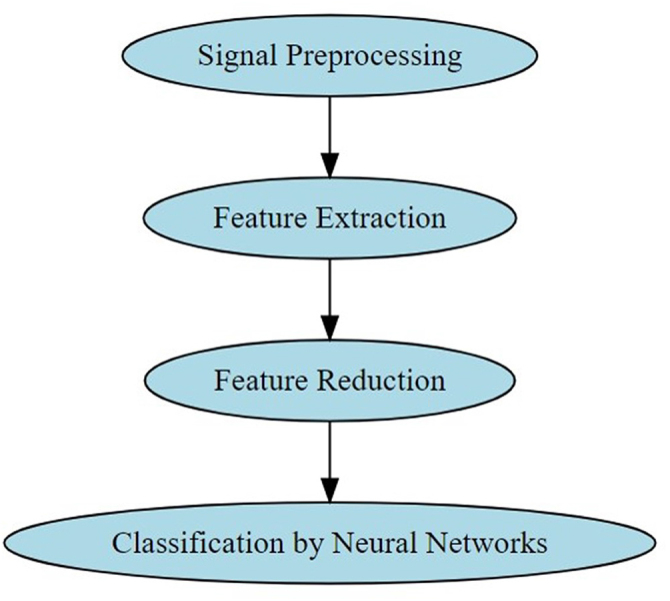
The working of proposed ensemble model.

Several AI models demonstrated 99% or more accuracy in beat and arrhythmia classification. Haseena *et al*^[23]^ developed an ensemble model by combining fuzzy expert rule-based system with deep neural networks for arrhythmia classification. The figure demonstrates the step-by-step implementation of the proposed AI model. The extracted features included ST segment duration, QT interval, PR interval, RR interval, R amplitude and QRS width. The model achieved an accuracy of 97% to 99.5% in classifying normal rhythms, LBBB, RBBB, PVC, APB, PB, VFW, and VEB. Figure 3 highlights the step-by-step functioning of the ensemble model.

In another study, an ensemble AI model^[24]^ was developed by combining CNN and LSTM techniques to classify variable length ECGs into normal heart rhythms, PVC, LBBB, APB and RBBB. LSTM models are used for learning temporal dependencies in sequential data like ECG signals, and are employed usually for tasks like arrhythmia detection and long-term cardiac monitoring to detect abnormal patterns overtime. Due to additional parameters, LSTM models are computationally expensive as compared to other deep networks^[25]^. The proposed model^[24]^ was trained and tested on PhysioNet public domain dataset and achieved 98.1% accuracy along with 97.5% sensitivity and 98.7% specificity.

### AI outperforms expert cardiologists

AI has outperformed expert cardiologists in arrhythmia classification. In a study, Hannun *et al*^[26]^ developed a DNN model to classify standard ECGs into 12 arrhythmic classes including AFib, AFL, AVB, SVT, VT, Wenckebach, trigeminy, bigeminy, ectopic atrial rhythm, idioventricular rhythm, junctional rhythm, etc. The algorithm achieved an average F1 score of 0.837, more than expert cardiologists’ 0.780 with an AUC of 0.977, sensitivity and specificity of >90%. In another study, LSTM model^[27]^ was developed to classify the above given 12 arrhythmia classes using standard 12-Lead ECGs. The DL technique outperformed interns, emergency physicians and cardiologists. Internists achieved an overall accuracy of 55%, emergency physicians 73%, cardiologists 83%, and LSTM model 90%.

The state-of-the art diagnostic AI algorithms have outworked physicians in detection of conduction abnormalities, structural abnormalities and MI in addition to arrhythmia detection. Hughes *et al*^[28]^ developed a CNN-based models on University of California, San Francisco dataset for the classification of 38 cardiac abnormalities. These abnormalities include PVC, PAC, AVB, LBBB, RBBB, QT prolongation, Wolff-Parkinson-White, ventricular hypertrophy, atrial enlargement, MI, etc. The model was validated against cardiologists’ diagnosis and consensus committee diagnosis comprising of electrophysiologists. The CNN model achieved an AUC of 0.91 and had comparable average F1 scores and higher sensitivity than cardiologists. Compared to another automated ECG system, MUSE, the current framework had higher F1 scores for all classes except SVT.

### AI for long-term cardiac monitoring

Holter monitors or insertable cardiac monitors (ICM) are used to monitor cardiac electrical activity for a longer time to reach quality diagnoses in certain diseases. It leads to the accumulation of large ECG data^[29]^. A DNN based model was developed^[30]^ on 1000 Holter recordings to detect the presence or absence of 5 cardiac abnormalities, i.e., VT, AFib, AFL, AT, AVB, and PVC. The algorithm was compared to traditional diagnostic platforms. Although no significant differences in sensitivity, accuracy and specificity were found except for AFib and VT but AI-analysis was 25% faster than conventional system which included electrophysiologists analysis of Holter recordings. The model demonstrated the feasibility of rapid, accurate analysis of Holter recordings with sensitivity and specificity ranging from 0.93 to 1 and 0.98 to 1, respectively.

**Figure 4.**
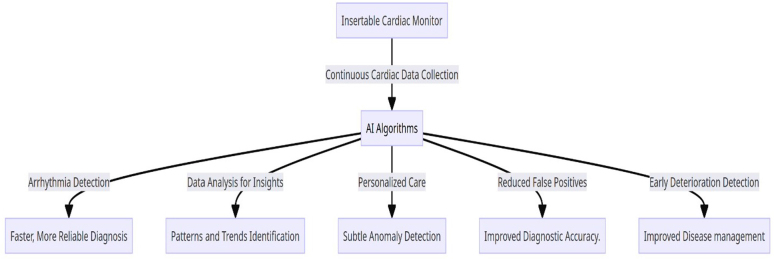
Applications of AI in ICM.

ICM is employed to detect various types of arrhythmias, risks of cardiac adverse events, personalized treatment and real-time monitoring of electrical activity of heart. Quartier *et al*^[31]^ employed AI algorithms for the analysis of ICM data collected over 23 months from 20 patients for arrhythmia detection. The AI model achieved 95.4% accuracy with 97.19% sensitivity, and 94.52% specificity for various classes of arrhythmias. It reduced the false positive arrhythmia by 98% overall. Figure 4 summarizes the AI’s role in ICM.

### AI-ECG for LVH

LVH is the consequence of myocardial hypertrophy. Although echocardiography is the gold standard for diagnosis but it can also be detected through ECGs as with increased myocardium, the amplitude for QRS complex also increases and electrical activity takes longer to transverse through the whole myocardium leading to QRS widening along with ST segment deviation. On ECG, a typical pattern in LVH includes ST segment deviation in opposite to QRS complex and T wave inversion. Several ECG criteria such as Cornell and Sokolow-Lyon have been developed. Lui *et al*^[32]^ developed a DL-augmented back-propagation neural network (BPN) model to detect LVH. The model extracted 24 features from the dataset followed by segmentation of individual beats in each case. In the end the BPN was trained using the extracted features. The model achieved 96.1% accuracy, 95.8% precision, 96.6% sensitivity and 95.6% specificity and outperformed all other ECG-LVH diagnostic criteria and other ML techniques.

### AI-ECG for asymptomatic ventricular dysfunction

ALVD is present in 3% to 6% of the individuals. AI can serve as a powerful tool for the detection of asymptomatic ventricular dysfunction. Attia *et al*^[33]^ developed a CNN-based diagnostic system to detect ALVD from standard 12 lead ECGs. The model was trained, tested and validated on the Mayo Clinic ECGs database and it achieved an accuracy of 85.7% along with a sensitivity 86.3%, specificity of 85.7%, and AUC 0.93. AI-positive for ALVD was found to be 4 times more at risk for symptomatic ventricular dysfunction.

### AI-ECG for HF

HFrEF is caused mainly by systolic dysfunction. AI-ECG models are of diagnostic importance for detection of HF from single lead ECGs as well as multiple lead ECGs. J Cho *et al*^[34]^ developed a CNN model to detect HFrEF (reduced ejection fraction is defined as <40%) from standard 12-Lead ECGs and single Lead ECGs as well. The model was trained, tested and validated on ECGs datasets from two hospitals. Upon internal and external validation of the model on standard 12-Lead ECGs, the model achieved an AUC of 0.913 and 0.961 respectively. With single-lead ECGs, the model achieved an AUC of 0.874 and 0.929 upon internal and external validation respectively demonstrating the diagnostic importance of single lead ECGs from the wearable devices for early screening of HF. Attia *et al*^[35]^ in a study developed a DL-algorithm to detect reduced ejection fraction from ECGs datasets obtained from Apple smartwatches with an AUC of 0.88. The study findings suggest that single lead ECGs from smartwatches obtained in non-clinical environment can be useful with AI integration.

### AI-ECG for CA

Stiff heart syndrome is also termed as cardiac amyloidosis (CA). AI can detect CA with higher accuracy. A study by Grogan *et al*^[36]^ demonstrated the potential of AI algorithms in CA screening from multiple Lead ECGs. The DNN model was trained, tested and validated on Mayo Clinic ECGs database. The 6-lead ECGs model achieved an AUC of 0.90 with precision of 0.85 for CA on single-lead ECGs, the model achieved an AUC of 0.86 with a precision of 0.78. This model could also predict CA progression 6 months earlier. In another study, an ML approach was utilized to detect multiple CVDs including CA, HTN, HCM, etc., with higher accuracy demonstrating the potential advantages of earlier screening.

### AI-ECG for valvular diseases

Valvular stenosis has a longstanding asymptomatic period and poor prognosis. However, AI-ECG systems can help with early screening of valve stenosis and regurgitation. Kwon *et al* developed DL models to detect aortic stenosis^[37]^ and mitral regurgitation^[38]^ with AUC of 0.816 to 0.884. Sensitivity maps reveal that DL algorithms analyze T waves from precordial leads to detect aortic stenosis while for mitral regurgitation analyzes both T-waves and P-waves in patients with mitral regurgitation.

### AI-ECG for MI detection

The advancements in AI especially the development of DL algorithms have enabled the invention of automated diagnosis systems with performance comparable to or better than physicians. In a study, Makimoto *et al*^[39]^ developed a 6-layered CNN model to detect MI from multi-lead ECGs. The proposed framework was trained, and tested on the PTB diagnosis ECG database which contains ECGs from 290 patients. The model achieved higher sensitivity, accuracy and specificity with 12-Leads ECGs as compared to 9-Lead ECGs and 6-Lead ECGs. The framework was also tested with 10 physicians for the recognition of MI with 72 ECGs. The model achieved a higher F1 score (0.83) and accuracy (81%) as compared to physicians.

### AI-ECG for risk prediction

AI-based ECG system has revolutionized the field of cardiology, particularly in the realm of risk prediction and early detection of cardiovascular events. These advance systems make use of various algorithms and data mining techniques to help identify patterns, and correlations, and trends essential for accurate risk prediction. These techniques make use of various algorithms and statistical models to analyze vast amounts of ECG data, electronic healthcare records, and other relevant medical information. The insights gained from this extensive analysis enable AI systems to refine their predictive models, improve diagnostic accuracy, and provide personalized recommendations for patient care. For instance, AI-ECG systems can reveal previously unnoticed relationships between specific ECG features and the likelihood of developing certain conditions.

One notable development during the COVID-19 pandemic was an ML model by Tsai *et al*^[40]^ designed to stratify high risk patients presenting to the emergency department (ED). This AI approach selected patients requiring ECGs for further diagnosis with an AUC of 0.89, demonstrating the role of AI in identifying high-risk individuals requiring necessary diagnostic attention. AI can stratify patients based on their risk of developing specific cardiac conditions by analyzing ECG data and other patient information. For instance, rECHOmmend, an ML model was developed to predict various structural abnormalities and cluster high-risk individuals. Trained on datasets from multiple sources, including ECGs from MUSE, Epic electronic health records, and echocardiograms from Xcelera, this model achieved an AUC of 0.91 with demographic details and ECGs as input^[41]^. AI models can be leveraged to predict the likelihood of future cardiac events, such as heart attacks or strokes, enabling healthcare providers to implement preventive measures and tailor monitoring and treatment plans individual risk profiles. This approach of personalized treatment not only enhances patient care but also improves the efficiency of healthcare delivery.

Moreover, AI ECG systems contribute to increase efficiency and accuracy in healthcare setting with their predictive capabilities. They can process large ECG datasets rapidly with higher accuracy, reducing the workload for clinicians and minimizing the risk of human error. For example, Akbilgic *et al*^[42]^ developed a CNN model to predict risks for heart failure (HF) solely from 12-lead ECGs with an AUC of 0.818, demonstrating comparable accuracy to conventional approaches such as FHS and ARIC risk calculators. Additionally, in HF patients with implanted cardiac sensors, ML technique can predict risk factors for rehospitalization^[43]^. These systems provide valuable diagnostic support, offering insights and recommendations based on extensive datasets and evidence-based algorithms. The model developed by Raghunath *et al*^[44]^ could predict all-cause mortality within one year from 12-lead ECGs with an AUC of 0.85, even when the ECGs were declared normal by cardiologists. By integrating data from various sources, including electronic health records (EHRs), wearable devices and other diagnostic tests, these systems provide a comprehensive risk assessment. Continuous learning from new data allows AI models to improve their predictive capabilities over time, ensuring that the insights they provide are up-to-date and accurate.

### Comparative analysis of different AI models

Table 2 compares various AI models discussed in this review.

**Table 2:** Compares various AI-models in terms of their strengths, limitations, and suitability

AI model	Strengths	Limitations	Suitability
1D-CNN	Efficient for time-series data, high accuracy in beat detection	Limited spatial feature extraction, requires large datasets	Single-lead or short-term ECG analysis
GRNN	Good generalization, works with small datasets	Computationally expensive, poor scalability.	Small datasets or specific beat classification tasks.
CNN	Robust feature extraction, High accuracy in multi-lead analysis	Computationally expensive, prone to overfitting	Multi-lead, large-scale ECG datasets, complex diagnosis.
RNN (LSTM/GRU)	Excellent for temporal sequence modeling, captures long-term dependencies.	High computational cost	Long-term ECG analysis, sequential data like continuous monitoring for arrhythmia.
Deep belief networks (DBN)	Can learn hierarchical features, good for semi-supervised learning.	Requires large datasets, slow training and limited scalability.	Tasks involving ECG feature extraction, useful in cases with both labeled and un-labeled data.
Ensemble models	Combines strengths of multiple models, high accuracy and robustness.	High-computational cost, slow inference due to multiple models.	Complex, multi-class ECG classification.
SVM	Strong generalization, works well for small datasets.	Limited to binary classification, less effective for large datasets.	Small datasets or binary classification tasks like distinguishing between normal and abnormal ECG signals.
STA-CRNN	Combines spatial and temporal attention for higher accuracy, robust to noisy data.	Complex to train, computationally expensive	Suitable for multi-class ECG classification, especially arrhythmia detection in noisy or large-scale datasets

### Navigating real-world implementation challenges and ethical issues

Although AI has made significant progress in ECG analysis, real-world implementation of AI-ECG systems poses numerous challenges, including computational demands, integration into existing healthcare systems, and the need for appropriate training of healthcare providers.

### Computational requirements:

AI models, particularly deep learning techniques like CNNs and RNNs, require significant computational resources, posing challenges in clinical environments, especially for real-time ECG analysis^[45]^. Training these models, particularly on large multi-lead ECG datasets, demands high processing power, often necessitating GPU acceleration. In real-time settings like ICUs or wearable devices, low-latency inference is crucial, but many healthcare facilities lack the necessary advanced hardware. Moreover, continuous monitoring using AI can increase energy costs, straining resources, especially in low- and middle-income countries. Solutions such as deploying models closer to the data source, and using techniques like model pruning and quantization, help reduce computational demands and enable deployment on less powerful hardware^[45]^.

### Integration into healthcare system

One of the hurdles to AI adoption in clinical settings is the difficulty in integrating AI models into existing healthcare systems, including electronic health records (EHRs) and medical devices. Healthcare systems are often built using platforms with varying levels of standardizations and compatibility. Integrating AI models into such systems requires ensuring that they can seamlessly interface with different data formats and communication protocols^[46]^. For instance, an AI system for ECG interpretation must be compatible with EHR systems to use patients’ data and add diagnostic results automatically. Moreover, AI models need to be compatible with existing medical devices such as Holter monitors, and wearable devices, which often use proprietary data formats^[47]^. Lack of standardization across device manufacturers can impede the integration of AI-driven solutions. Also, AI models rely on large datasets for training and continuous improvement. However, accessing and sharing patient ECG data across institutions is often restricted due to privacy regulations such as HIPAA in United States and GDPR in Europe, creating barrier to AI implementation. These hurdles can be overcome by ensuring that AI models are built to comply with healthcare data exchange standards like HL7 (Health level 7) and FHIR (Fast Healthcare Interoperability Resources) which is crucial facilitating communication between different healthcare systems. Additionally, use of application programming interfaces (APIs) and intermediary solutions can bridge the gap between AI models and existing healthcare systems, enabling smoother data exchange^[46]^.

### “Black-Box” AI algorithms

The implementation of AI in clinical settings also requires training healthcare professionals, i.e., physicians, nurses and other healthcare staff that may be unfamiliar with AI technologies leading to skepticism or improper use. Physicians are often hesitant to trust AI systems, especially when models function as “black boxes” without offering clear explanations^[48]^. Clinicians must be reluctant to rely on AI-based diagnostic tools if they cannot easily understand how the model arrived at a particular diagnosis. This lack of transparency, especially in DL models, raises concerns about the accountability in cases of misdiagnosis or errors. This question needs to be addressed “Who is responsible when an AI model makes a mistake?”

Implementation of AI literacy programs for healthcare professionals is essential. Training should focus on helping clinicians understand the strengths and limitations of AI models, how to interpret AI-driven results, and how to integrate these results into their clinical decision-making process. Additionally, the development of explainable AI tools can help address the “black box” issue by making AI model decisions more interpretable for healthcare professionals. Providing clinicians with user-friendly interfaces that clearly present AI outputs, along with confidence scores and visual explanations of the part of ECG that led to a particular classification, can improve trust and usability. Encouraging human-AI collaboration rather than full reliance on AI can build clinician trust. AI systems designed as decision support tools rather than autonomous diagnostic tools, where the final decision still rests with the healthcare provider, will allow clinicians to use AI as “second opinion” without feeling that their role is diminished. Moreover, collaborations of AI experts, data scientists and clinicians will boost the integration of AI in clinical workflow^[49]^.

### Regulatory and ethical challenges

AI models for clinical use must meet stringent regulatory requirements for approval such as those set by the FDA (Food and Drug Administration) or the European Medicines Agency (EMA). Working closely with regulatory bodies during the development of AI systems can help streamline the approval process. This would also ensure that AI tools are built in compliance with safety standards and are validated through robust clinical trials. Ensuring data privacy in AI models that process patient information is a major concern. In addition to complying with privacy regulations, hospitals must also guard against cyberattacks that could compromise patient data and AI systems^[48]^.

### Noises in ECG datasets

Noise can be effectively handled with a combination of preprocessing techniques and noise-resilient model architectures. Preprocessing involves techniques such as bandpass filtering, which removes high- and low-frequency noises, by breaking down the ECG signals into different frequency components, filtering out noise at specific levels. Independent component analysis is another method that helps isolate the true ECG signals from noise, particularly useful when analyzing data from multiple ECG leads. In addition to pre-processing, noise-robust AI models are designed to handle noisy input data more effectively. Ensemble models, which combine multiple algorithms such as CNNs (for spatial analysis) and LSTMs (for temporal patterns), offer a more comprehensive analysis by balancing the strengths of each model. This makes them more resilient to the inconsistencies caused by noise. Furthermore, attention mechanisms embedded in these models help focus the AI’s analysis on the most relevant parts of the ECG signals, essentially filtering out noisy segments by selectively highlighting the important features for diagnosis.^[11]^

### Training dataset generalizability limitations

Most of the AI algorithms are trained on limited ECG datasets obtained from electronic healthcare records of one institute or more institute of a city or a country. This may limit the generalizability of the algorithms and lead to algorithm bias. Therefore, there is the need for preparation of diversified databases available for everyone to ensure generalizability in the AI algorithm being developed in every corner of the world^[50]^.

### Multi-modal AI systems

Most of the AI-ECG systems are developed on ECG input but incorporation of risk indicators, demographics, and other tests as additional input improves the performance of the AI systems. Therefore, multimodal AI models must be developed for accurate diagnosis and personalized treatment plans^[51]^.

## Conclusion

The applications of AI-ECG have seen remarkable advancements in recent years. DL models like CNN have not only enabled automated detection and classification of several cardiac abnormalities but also outperformed cardiologists. AI can detect AFib, AFL, VTA, LVH, MI, ALVD, etc., with high accuracy and also identify high-risk individuals and predict cardiac adverse events from multiple lead ECGs. However, there are still challenges to address before widespread clinical implementation including improved interpretability, diversified training datasets, cost effectiveness evaluation. Overall, AI-ECG systems hold promise to improve the efficiency, accuracy and sensitivity of ECG interpretations to guide screening and personalized management of cardiac abnormalities. More research is required to develop robust and generalizable AI algorithms to integrate into clinical workflow. Close collaboration between cardiologists, data scientists and software engineers will be key to realizing AI’s full potential in advance cardiac care.
